# Lignin degradation by a novel thermophilic and alkaline yellow laccase from *Chitinophaga* sp.

**DOI:** 10.1128/spectrum.04013-23

**Published:** 2024-05-07

**Authors:** Bárbara Bonfá Buzzo, Natália Sarmanho Monteiro Lima, Pâmela Aparecida Maldaner Pereira, Elisângela Soares Gomes-Pepe, Camila Cesario Fernandes Sartini, Eliana Gertrudes de Macedo Lemos

**Affiliations:** 1 Department of Agricultural, Livestock and Environmental Biotechnology, São Paulo State University Júlio de Mesquita Filho, Jaboticabal, São Paulo, Brazil; 2 Molecular Biology Laboratory, Institute for Research in Bioenergy (IPBEN), Jaboticabal, São Paulo, Brazil; 3 Agricultural Microbiology Graduate Program at UNESP, Jaboticabal, São Paulo, Brazil; Dominican University New York, Orangeburg, New York, USA

**Keywords:** yellow laccase, extremophile, lignin

## Abstract

Laccases (EC 1.10.3.2) are oxidoreductases that belong to the multicopper oxidase subfamily and are classified as yellow/white or blue according to their absorption spectrum. Yellow laccases are more useful for industrial processes since they oxidize nonphenolic compounds in the absence of a redox mediator and stand out for being more stable and functional under extreme conditions. This study aimed to characterize a new laccase that was predicted to be present in the genome of *Chitinophaga* sp. CB10 – Lac_CB10. Lac_CB10, with a molecular mass of 100.06 kDa, was purified and characterized via biochemical assays using guaiacol as a substrate. The enzyme demonstrated extremophilic characteristics, exhibiting relative activity under alkaline conditions (CAPS buffer pH 10.5) and thermophilic conditions (80–90°C), as well as maintaining its activity above 50% for 5 h at 80°C and 90°C. Furthermore, Lac_CB10 presented a spectral profile typical of yellow laccases, exhibiting only one absorbance peak at 300 nm (at the T2/T3 site) and no peak at 600 nm (at the T1 site). When lignin was degraded using copper as an inducer, 52.27% of the material was degraded within 32 h. These results highlight the potential of this enzyme, which is a novel yellow laccase with thermophilic and alkaline activity and the ability to act on lignin. This enzyme could be a valuable addition to the biorefinery process. In addition, this approach has high potential for industrial application and in the bioremediation of contaminated environments since these processes often occur at extreme temperatures and pH values.

**IMPORTANCE:**

The characterization of the novel yellow laccase, Lac_CB10, derived from *Chitinophaga* sp. CB10, represents a significant advancement with broad implications. This enzyme displays exceptional stability and functionality under extreme conditions, operating effectively under both alkaline (pH 10.5) and thermophilic (80–90°C) environments. Its capability to maintain considerable activity over extended periods, even at high temperatures, showcases its potential for various industrial applications. Moreover, its distinctive ability to efficiently degrade lignin—demonstrated by a significant 52.27% degradation within 32 h—signifies a promising avenue for biorefinery processes. This newfound laccase's characteristics position it as a crucial asset in the realm of bioremediation, particularly in scenarios involving contamination at extreme pH and temperature levels. The study's findings highlight the enzyme's capacity to address challenges in industrial processes and environmental cleanup, signifying its vital role in advancing biotechnological solutions.

## INTRODUCTION

Multicopper oxidases (MCOs) constitute a superfamily of oxidoreductases that play essential catalytic roles and require copper atoms as cofactors. Within the functional diversity of MCOs, distinct subclasses, such as ascorbate oxidases (EC 1.10.3.3), bilirubin oxidases (EC 1.3.3.5), ferroxidases (EC 1.16.3.1), and laccases (EC 1.10.3.2), stand out (1).

Laccases are the largest subfamily among MCOs. The active site of these materials is composed of four copper atoms distributed across three distinct centers, exhibiting variations in their paramagnetic resonance signals. These centers are categorized as type 1 copper centers (T1), known as blue copper, with an absorption band at approximately 600 nm; type 2 copper centers (T2), corresponding to normal copper; and type 3 copper centers (T3), termed coupled binuclear centers, which absorb light at approximately 300 nm. These centers are crucial for the efficient catalytic performance of the enzyme (2). Most laccases display two absorption peaks at the copper centers T1 (600 nm) and T2/T3 (300 nm), classifying them as blue laccases (3).

However, there are specific cases where some laccases are described as having only one absorption peak at approximately 300 nm, associated with the T2/T3 centers. These laccases are referred to by some researchers as yellow laccases and by others as white laccases. Both denominations are acceptable for characterizing laccases with such spectral characteristics (4, 5).

The change in the spectrum of laccases has not been well defined to date, but the absence of blue coloring may be related to low-molecular-weight phenols derived from lignin during lignocellulose degradation, a theory first proposed by Leontievsky et al. (5). Recent theories suggest that the transition from blue to yellow laccases may include changes in oxidation levels (6), structural characteristics (7), glycosylation states (8), and interactions with metal ions (6). In addition to the spectral differences between blue and yellow laccases, the latter are capable of oxidizing nonphenolic compounds in the absence of a redox mediator (5) and stand out for their high stability and functionality under extreme conditions (5, 9).

In general, laccases are widely distributed in fungi, bacteria, plants, and insects, and due to their broad substrate specificity, laccases have been extensively explored in various biotechnological applications, such as delignification, dyeing, degradation, and decolorization of textile dyes; bioremediation of xenobiotics and organic pollutants; biobleaching; biofuel production; and biosensing (6, 7).

Most of the described laccases are fungal in origin and include those from *Panus tigrinus, Phlebia radiata*, and *Phlebia tremellosa* (5); *Stropharia aeruginosa* (10); *Ganoderma fornicatum* (11); *Pleurotus ostreatus* (9); *Leucoagaricus naucinus* (12); *Daedalea flavida* (13); *Trametes* sp. F1635 (14); and *Sclerotinia sclerotiorum* (8). However, studies have shown that some bacterial laccases have great potential for industrial use due to their extremophile properties, such as high thermal and pH stability, rapid growth rate, and ability to be maintained under ideal environmental conditions (15, 16). Some characterized bacterial laccases include those from *Azospirillum lipoferum* (17), *Aquifex a*eolicus VF5 (18), *Sinorhizobium meliloti* (19), *Bacillus tequilensis* SN4 (20), and *Alcaligenes faecalis* (21).

Lignin is the most abundant component of lignocellulosic biomass, and despite its potential, the industry considers it a byproduct due to its transformation difficulties (22). Recently, publications reporting various lignin degradation compounds using whole-cell bacterial laccase strains under different pH conditions have been published (23). Recognizing the importance of finding new bacterial laccases for industrial application, the present work introduces a new yellow laccase from *Chitinophaga* sp. CB10 that has been proven to be active and stable at high temperatures and basic pH and has promising action in lignin degradation.

## MATERIALS AND METHODS

### Organism

The microorganism used here, *Chitinophaga* sp. CB10, was isolated from a consortium obtained from soil with a high biomass degradation rate and cultivated on sugarcane bagasse and carboxymethyl cellulose (CMC). In a previous study, the genome of this microorganism was partially sequenced (24, 25) and is deposited under the accession number MLAV00000000 in the National Center for Biotechnology Information (NCBI) GenBank, which is also part of the internal collection of genomes and metagenomes of Laboratory of Microorganism and Plant Biochemistry, LBMP.

### Construction of the expression vector pETSUMO-LacCB10

The Lac_CB10 gene consists of 2,187 base pairs and was amplified via PCR using the primers lac-F1 (5′-TA TGGATCCCCCAAAACCGTTCGGTACGACCTC-3′) and lac-R1 (5′-ATATAAGCTTAATAACTCAACGTAATAC-3′). The PCR cycling parameters used to amplify the gene were as follows: initial denaturation at 95°C for 2 min; 30 cycles of denaturation at 95°C for 45 s and annealing at 60°C for 45 s; and extension of the gene by DNA polymerase at 68°C for 2.5 min. The PCR products were analyzed using a 1% (wt/vol) agarose gel. The PCR products were subsequently cloned and inserted into the pET SUMO vector following the Champion pET SUMO TA expression system protocol (Invitrogen, USA), resulting in the construction of pETSUMO-LacCB10.

Notably, the Lac_CB10 gene contains a signal peptide predicted by SignalP and was cloned and inserted into the pET-SUMO vector without including the corresponding signal peptide sequence.

### Transformation of *Escherichia coli* ER2265 competent cells with pETSUMO-LacCB10

*Escherichia coli* ER2265 chemically competent cells were transformed with the pETSUMO-LacCB10 plasmid, inoculated onto agar plates containing Luria–Bertani (LB) medium supplemented with 50 µg/mL kanamycin and incubated at 37°C for 16 h. Clones were confirmed by colony PCR using SUMO forward and reverse primers and stored in 20% (vol/vol) glycerol at −80°C.

### Expression, extraction, and purification of the recombinant enzyme

For the production of the recombinant enzyme, 50 mL of liquid LB medium supplemented with 50 µg/mL kanamycin was used to culture an isolated colony of the pETSUMO-LacCB10 plasmid transformed into *E. coli* ER2265 under shaking at 200 rpm for 16 h at 37°C to prepare the preinocula. The inoculum was prepared with 1 L of LB medium supplemented with 50 µg/mL kanamycin, 1% (vol/vol) of the preinoculum was added, and the mixture was shaken at 200 rpm at 37°C until it reached an OD_600_ between 0.4 and 0.6 nm. The mixture was induced with 0.9 mM IPTG and cultivated at 200 rpm for 4 h at 28°C. After induction, the cells were centrifuged at 6,400 × *g* (Sorvall RC5C; Kendro Lab Products, Ashville, NC, USA) and 4°C for 30 min and resuspended in 50 mM K2HPO4 buffer (pH 7.8), 400 mM NaCl, 100 mM KCl, 10% (vol/vol) glycerol, 0.5% Triton X-100, and 3 mM PMSF. The cells were lysed by ultrasonication (Branson Sonifier 250, Branson, CT, USA) with a duty cycle of 20% and 12 cycles of 15 pulses and intervals of 15 s. The lysed cells were centrifuged for 20 min at 6,400 × *g* at 4°C to obtain the extracts.

The soluble extract was incubated for 1 h at 4°C under stirring with 3.5 mL of Ni 2+–NTA resin (Qiagen, Hilden, Germany), which was previously equilibrated with the same buffer used in the enzymatic extraction step. After this incubation, the sample composed of the resin and bound extract was subjected to an imidazole gradient from 20 mM to 1 M. The protein of interest was eluted using the same extraction buffer containing 500 mM imidazole.

The recombinant protein was subsequently concentrated using an Amicon Ultra15 filter with a molecular cutoff of 10 kDa (MWCO 10) (Merck Millipore, Billerica, MA, USA) and subjected to additional chromatography to remove contaminants and estimate the molecular weight and oligomeric state of the compounds. Molecular exclusion chromatography was conducted using an AKTA Pure (GE Healthcare Bio Sciences, Uppsala, Sweden) and a Hiload 16/600 Superdex column (GE Healthcare Bio Sciences, Uppsala, Sweden) at a flow rate of 1 mL/min, an injection of 2 mL and a pressure of 0.42 mPa. The parameters of flow, injection, and fractionation were controlled using UNICORN 5.0 software (Amersham Bioscience Co., Piscataway, NJ, USA). The protein concentration was estimated using the Bradford assay with bovine serum albumin as a standard at 595 nm (26).

### Copper binding and enzyme concentration determination

The purified recombinant protein was incubated with 1 mM CuSO4 for 6 h to allow copper to bind to the copper-binding sites, and thus, the active holoenzyme was bound to its cofactor (copper). After incubation, the excess copper was removed using 50 mM potassium phosphate buffer (pH 7.8) supplemented with 1 mM ethylenediaminetetraacetic acid (EDTA), followed by five additional washes with the same buffer without EDTA, in accordance with the modified protocol of Machczynski et al. (27). The washes were completed using an Amicon Ultra15 filtration device with a 10 kDa molecular weight cutoff (MWCO 10) (Merck Millipore, Billerica, MA, USA). The protein concentration was estimated using the Bradford assay with bovine serum albumin as the standard at 595 nm (26).

### SDS-PAGE, Western blotting, and zymogram

The purity of the enzymes was confirmed by polyacrylamide gel electrophoresis with sodium dodecyl sulfate (SDS‒PAGE), which was carried out following the protocol of Laemmli (28). The enzyme was purified, and its purity was assessed through polyacrylamide gel electrophoresis with a 5% (wt/vol) stacking gel and a 7% (wt/vol) resolving gel stained with Coomassie Brilliant Blue R-250. The purified enzyme was also subjected to Western blot analysis, where the 7% (wt/vol) SDS polyacrylamide gel containing the enzyme sample was transferred to a polyvinylidene difluoride membrane (PVDF, Thermo Fisher Scientific) with a porosity of 0.45 µm using a Mini Trans-Blot system (Bio-Rad Laboratories, Hercules, CA, USA) in 10 mM CAPS buffer at pH 11.0 containing 10% (vol/vol) methanol at 90 V for 45 min at 4°C. Nonspecific sites were blocked with phosphate-buffered saline (PBS), 0.02% Tween 20, and 5% skim milk powder (Molico) (1:1,000 dilution). The membrane was incubated with an anti-polyhistidine monoclonal antibody (H1029; Sigma, Saint Louis, MO) and a secondary peroxidase-conjugated anti-mouse IgG antibody (A9044; Sigma, Saint Louis, MO, USA). Detection was carried out in the presence of 3,3′-diaminobenzidine tetrahydrochloride in 15 mL of PBS (pH 7.6) containing 12 µL of 30% H_2_O_2_.

The laccase activity was assessed through a zymogram test. The protein bands were separated using a nondenaturing electrophoretic assay (7% PAGE) at 4°C and 100 V and subsequently incubated in the presence of catechol (10 mM) and guaiacol (0.02%(vol/vol) ) substrates in CAPS buffer at pH 10.5.

### UV-Visible analysis

UV‒Vis spectra were recorded for samples of the Lac_CB10 holoenzyme and apoenzyme using a spectrophotometric reading range of 200–900 nm with a 5-nm interval and a UV‒Vis spectrophotometer (Multiscan GO; Thermo Fisher Scientific, Waltham, MA, USA). The spectra of Lac_CB10 were compared to those of a confirmed blue laccase, as described by Lima et al. (29).

### Determination of the optimal pH and temperature parameters

The laccase activity was determined by reading the absorbance values obtained after the oxidation of the substrate guaiacol (420 nm). The reactions were carried out in microplates with a total volume of 100 µL, consisting of 20 µL of buffer (20 mM), 1.24 µL of substrate (1 mM), and 10 µL (35,82 µg/µL) of the enzyme. The effect of pH was assessed in the range of 3.0–12.0 using the following buffers: sodium acetate (pH 3.0, 4.0, 5.0, 5.5, and 6.0); MES (pH 5.5, 6.0, and 6.5); PIPES (pH 6.0, 6.5, 7.0, and 7.5); MOPS (pH 6.5, 7.0, 7.5, and 8.5), and 8.5; Tris-HCl (pH 6.5, 7.5, and 8.0); Tris-HCl (pH 6.5, 7.0, 7.5, 8.0, and 8.5); TAPS (pH 8.0 and 9.0); Ampol (pH 9.0, 9.5, and 10.0); CAPS (9.5, 10.0, 10.5, and 11.0); glycine (pH 8.5, 9.0, 9.5, 10.0, and 10.5); sodium bicarbonate (pH 9.0, 9.5, 10.0, 10.0, 10.5, 11.0, and 12.0); and potassium phosphate (pH 11.0, 11.5, and 12.0).

The pH parameters were assessed after 16 h at 37°C because the substrate did not immediately change in color when added to the laccase reaction, regardless of pH or temperature. Only after 16 h was it possible to observe the characteristic brown coloration resulting from substrate oxidation.

The optimal temperature was determined at the optimal pH as indicated in the previous test and assessed at temperatures of 10°C, 15°C, 25°C, 30°C, 37°C, 40°C, 50°C, 60°C, 70°C, 80°C, and 90°C for 1 h. The thermostability of the enzymes was evaluated by preincubating them for 5 h at 80°C and 90°C. After every 30 min, a 10-µL aliquot was taken and added to a mixture containing the buffer at the ideal pH and the guaiacol substrate, and the reaction was carried out at the optimal temperature, as indicated in the previous test.

The tolerance of the enzymes, including SDS, Triton X-100, and Tween 80, to detergents was also evaluated at concentrations of 0.5% and 1% (vol/vol), as was the tolerance to EDTA and DTT at concentrations of 1 mM, 5 mM, and 10 mM. Solvent tolerance was analyzed with methanol and acetone at concentrations of 1%, 2%, and 5% (vol/vol), and the effects of salts and metal ions were assessed with CuSO_4_, MgSO_4_, NiCl_2_, CaCl_2_, NaCl_2_, CoCl_2_, KCl, FeSO_4_, MnSO_4_, Al_2_(SO_4_)_3_, Li_2_SO_4_, AgNO_3_, BaCl_2_, ZnSO_4_, CdCl_2_, and KI at 2 mM, following the optimal parameters of pH and temperature.

### Lignin degradation by Lac_CB10 whole cell

The assays were conducted using *E. coli* cells harboring Lac_CB10. A preinoculum was prepared as described in Section Expression, extraction, and purification of the recombinant enzyme and subsequently added to flasks containing 0.4 mg/mL alkaline lignin (AL) and LB medium. The assay proceeded for 36 h under agitation at 150 rpm and a temperature of 55°C. Two treatments were performed: one without any inducer, consisting only of the transformed cells, and another with copper (0.25 mM) as an inducer. The degradation calculation was performed following the description in (30):

Degradation (%) =Ai − Af × 100

where Ai is the initial absorbance and Af is the final absorbance.

### SEM analysis of lignin degradation

The two lignin treatments, one without an inducer and the other with an inducer, were examined using scanning electron microscopy (SEM; ZEISS EVO MA10) to determine potential structural changes in the lignin. The samples were first lyophilized and subsequently mounted on carbon tape. Additionally, they were coated with gold to facilitate viewing and imaging.

### Statistical analysis

All experiments conducted in this study were performed with statistical replicates (triplicates). The data were analyzed using analysis of variance (ANOVA) with the Tukey multiple comparisons test at a 5% probability level.

## RESULTS

### Cloning and production of the recombinant laccase

**Fig 1:**
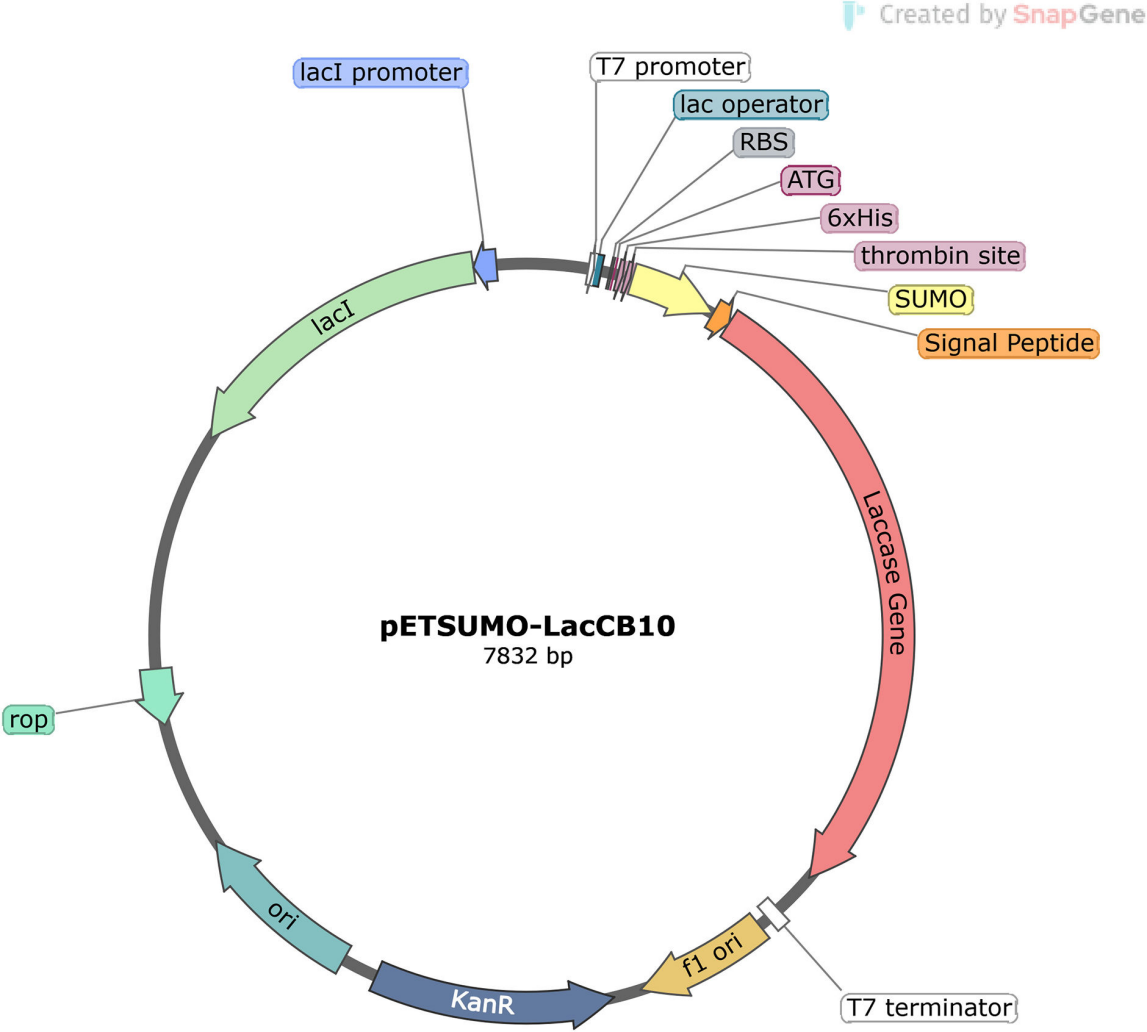
Schematic representation of the pET-SUMO plasmid containing the Lac_CB10 insert. From this expression system, cloning was performed to enable expression in the *E. coli* ER2265 strain.

The open reading frame of the Lac_CB10 laccase gene comprises 2,184 nucleotides and encodes a protein of 728 amino acids with a predicted molecular weight of 94 kDa. The gene was cloned and inserted into the pETSUMO (Fig. 1) expression vector and transformed into competent *E. coli* ER2265 cells. Positive colonies were identified by selection with the antibiotic kanamycin, the resistance gene present in the pETSUMO vector.

### Enzyme expression and purification

**Fig 2:**
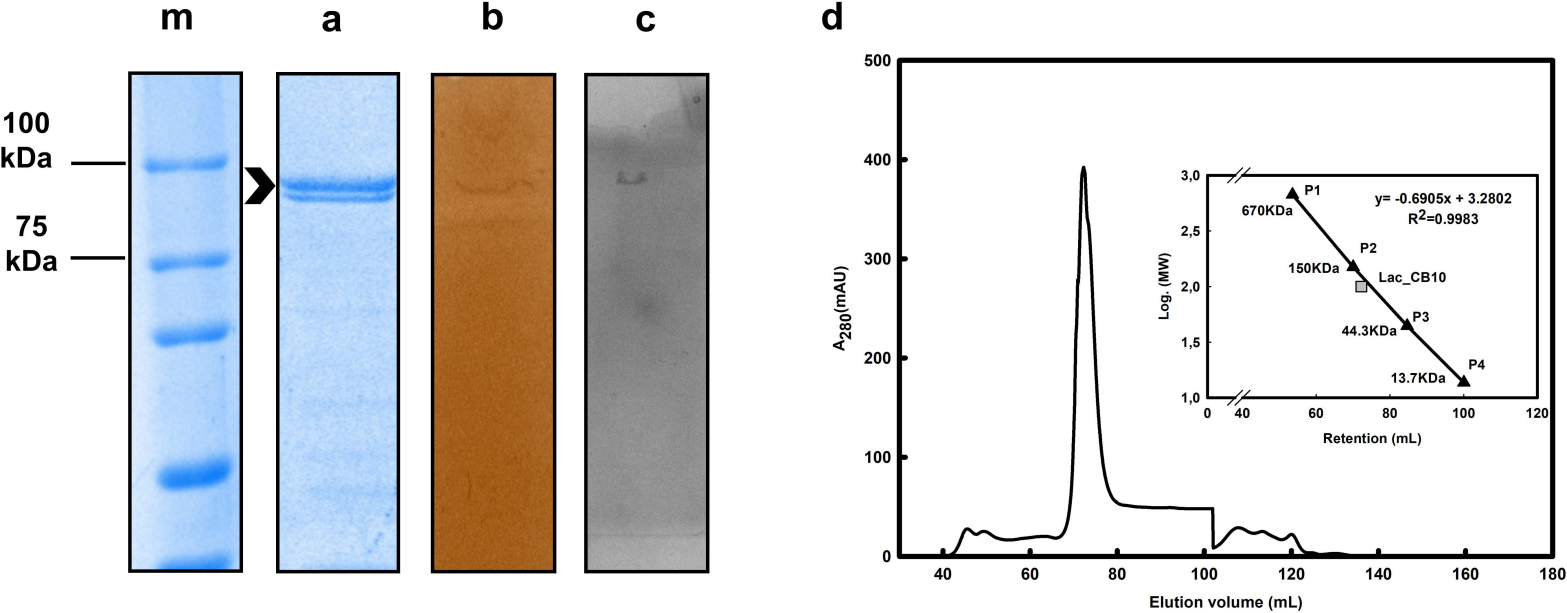
Extraction and purification of Lac_CB10. (M) Molecular mass marker. (A) Purified Lac_CB10 in 7% SDS-PAGE gel. (B) Native PAGE gel stained with 10 mM catechol in CAPS buffer pH 10.5. (C) Native PAGE gel stained with 0.02% guaiacol in CAPS buffer pH 10.5. (D) Chromatographic profile of purified laccase by gel filtration. Estimation of molecular mass of Lac_CB10 by gel filtration based on linear correlation. Elution of molecular mass standard of Lac_CB10 and protein standards versus their logarithmic molecular mass values. P1–P4: Proteins used as molecular mass standards: ρ-aminobenzoic acid (pABA) (0.13 kDa), ribonuclease A (13.7 kDa), albumin (43 kDa), bovine thyroglobulin (670 kDa), γ-globulin (150 kDa), and Protein Standard Mix 15 ± 600 kDa, Sigma, St. Louis, MO, USA.

Optimized expression conditions at 28°C and 200 rpm for 4 h resulted in an enzymatic yield of 10.92 mg/L culture. In the first purification step, recombinant laccase with a histidine tail (6× His) and nickel Ni-NTA resin (Qiagen Venlo Netherlands) were used to isolate the Lac_CB10 protein, and these fractions were analyzed via SDS‒PAGE (Fig. 2). SDS‒PAGE analysis revealed a single purified band with a molecular mass of approximately 94 kDa, which was consistent with the mass predicted by the ProtParam program for the fusion protein. Molecular exclusion chromatography was used to predict the mass of the Lac_CB10 protein in its native apoenzyme configuration through linear regression analysis of the ratio of the column’s void elution volume and the logarithm of the molecular mass in the same elution volume (Ve/Vo) obtained for samples of a commercial molecular weight standard. The estimated molecular mass was 100.06 kDa.

### Spectral properties of Lac_CB10

**Fig 3:**
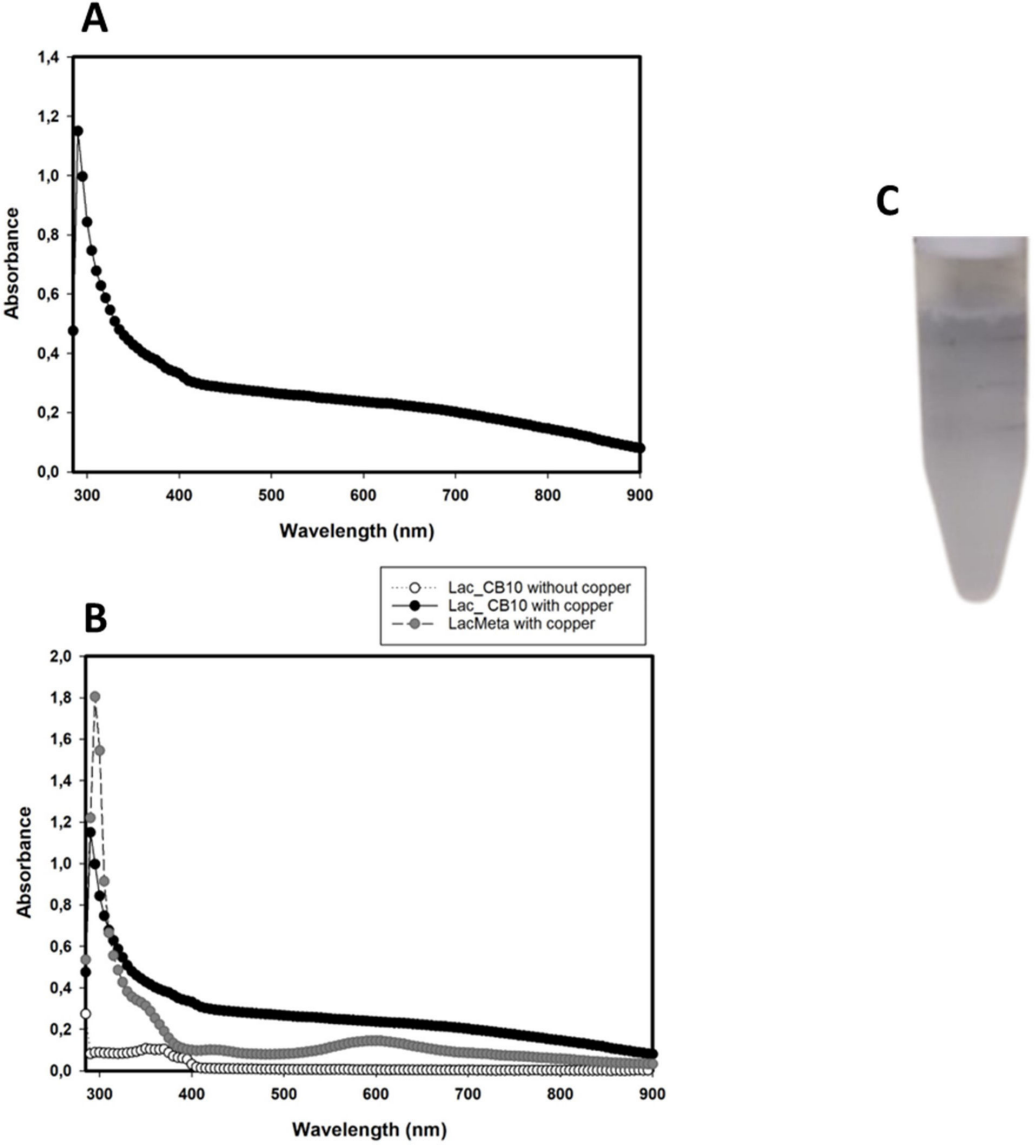
UV-Vis spectrum of Lac_CB10 apoenzyme and holoenzyme. (A) UV-vis spectrum of Lac_CB10 in its holoenzyme form, with an absorption peak near 330 nm. (B) UV-vis spectrum of Lac_CB10 (black circles) in its apoenzyme form (without any absorption peak) and of the holoenzyme compared to the blue laccase LacMeta (gray circles), showing their absorption peaks at 330 nm and 600 nm.

The UV‒Vis spectra of the Lac_CB10 enzyme were measured in apoenzyme and holoenzyme states before and after incubation with CuSO_4_, respectively. As expected, the apoenzyme showed no absorption at the measured wavelengths. Conversely, after the laccase had captured the copper atoms and filled its binding sites, there was an absorption peak at approximately 300 nm and no peak at 600 nm (Fig. 3A and B).

Interestingly, the authors found atypical patterns of substrate oxidation that resulted in unconventional colors for various tested substrates, which led to the proposition of a new class of laccases, the “Rainbow” laccases (8). Taken together, these data indicate that there is still much to be discovered about the mechanisms of action and physicochemical characteristics of laccases, improving the still confused classification system of these enzymes.

### Enzymatic characterization: pH, temperature, and thermostability

**Fig 4:**
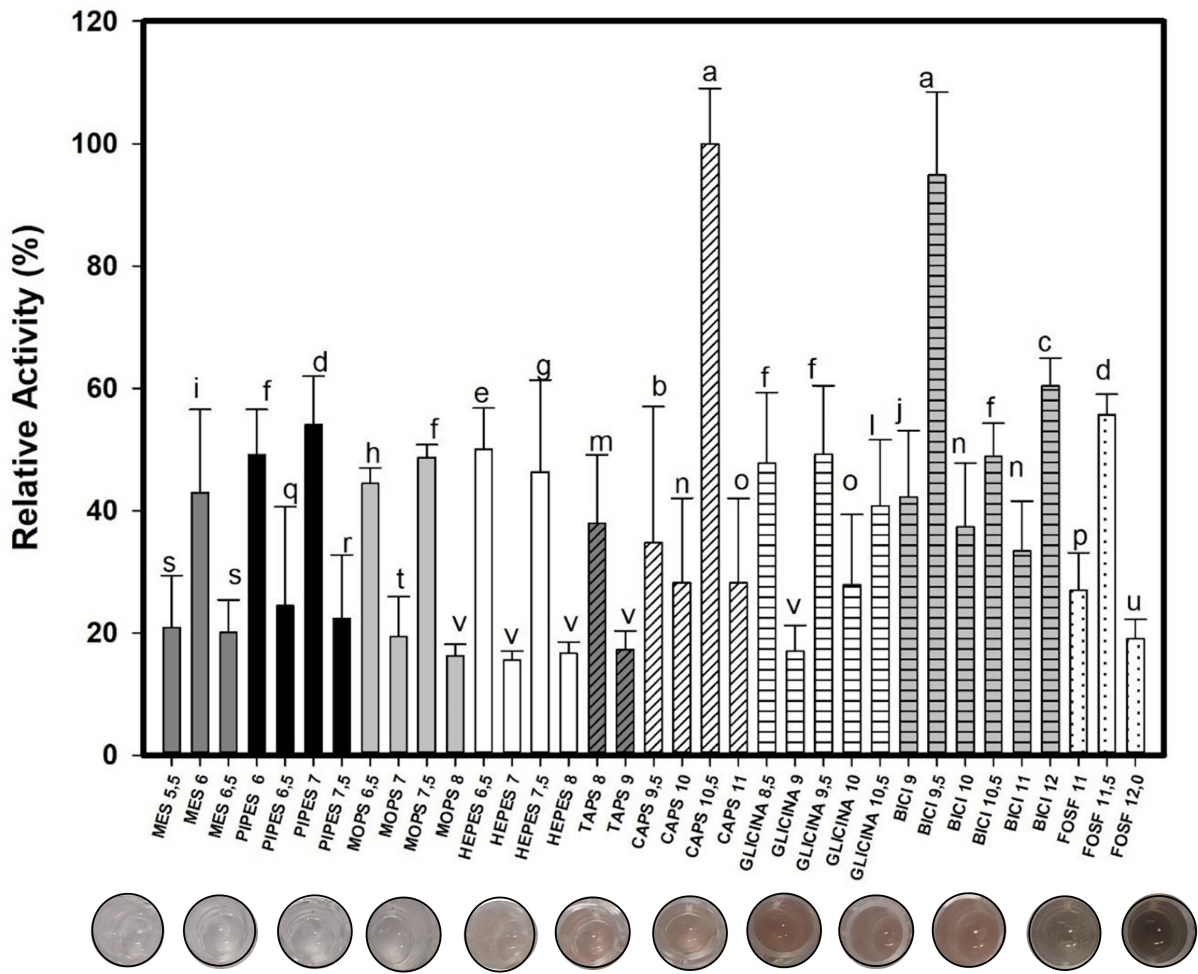
Effect of pH on the activity of Lac_CB10. Enzymatic activity was performed using the following 20 mM buffers: MES (pH 5.5, 6.0, and 6.5); PIPES (pH 6.0, 6.5, 7.0, and 7.5); MOPS (pH 6.5, 7.0, 7.5, and 8.0); HEPES (pH 6.5, 7.0, 7.5, and 8.0); TAPS (pH 8.0 and 9.0); CAPS (pH 9.5, 10.0, 10.5, and 11.0); Glycine (pH 8.5, 9.0, 9.5, 10.0, and 10.5); sodium bicarbonate (pH 9.0, 9.5, 10.0, 10.5, 11.0, and 12.0); and potassium phosphate (pH 11.0, 11.5, and 12.0). The tests were conducted with three replicates. Values with the same letter do not differ statistically, according to the ANOVA and Tukey test, with a 5% probability.

pH evaluations revealed that, after 16 h of incubation at 37°C with guaiacol, Lac_CB10 exhibited the greatest activity at relatively basic pH values, reaching 100% of its activity in CAPS buffer at pH 10.5. Guaiacol is a phenolic substrate that turns brown when oxidized by an enzyme, and it was interesting to note that as soon as the substrate was reacted with laccase, there was no change in color at any of the tested pH values at room temperature or 37°C until after 16 h. Figure 4 shows that as the pH increases, the substrate is more oxidized by the enzyme; that is, its color becomes more brown.

**Fig 5:**
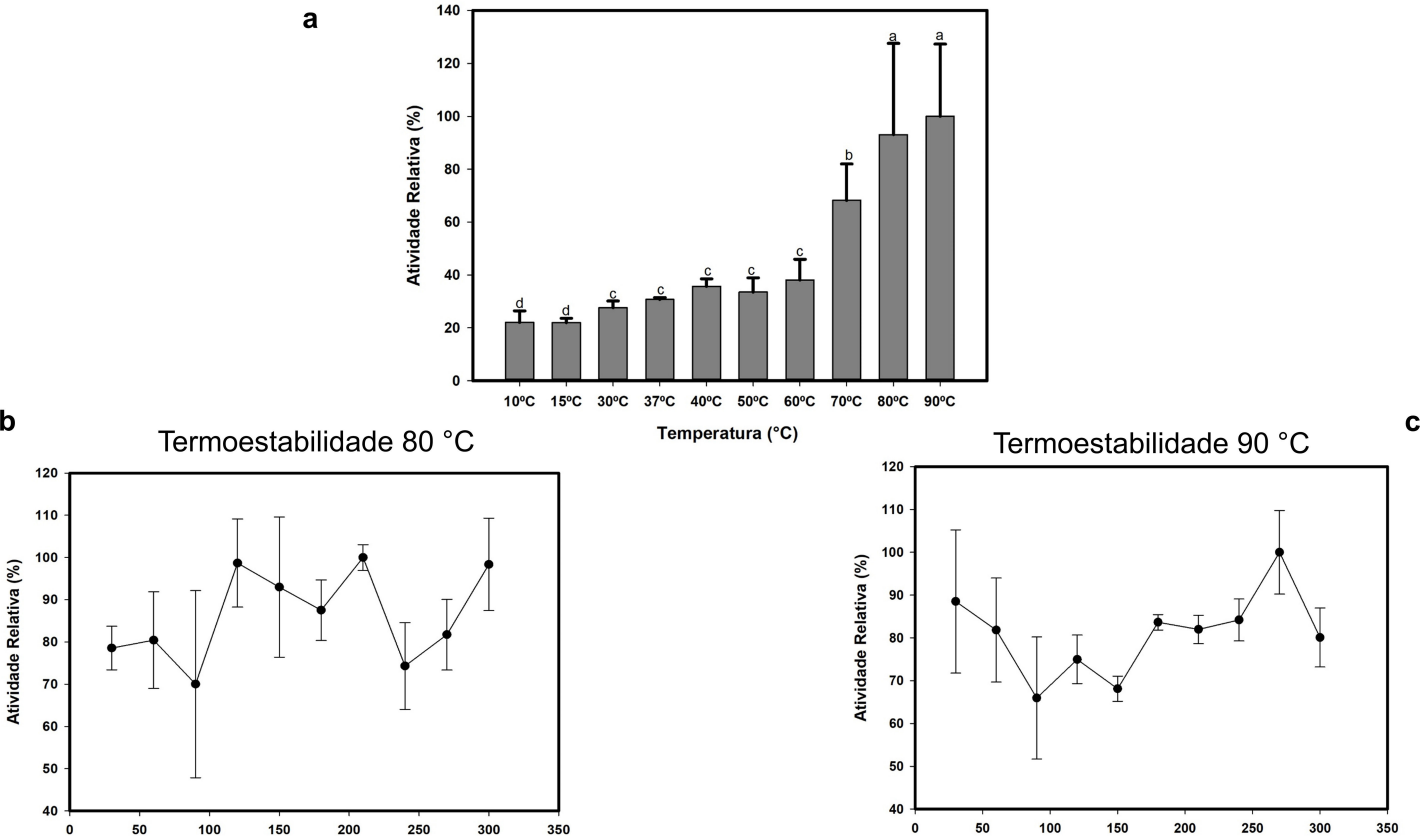
Effects of temperature on Lac_CB10 Activity. (A) The enzymatic activity was assessed at temperatures ranging from 10°C to 90°C in a 20 mM ampol buffer (pH 10.0). (B and C) The thermostability tests were conducted in a 20 mM CAPS buffer (pH 10.5) over 5 h at 80°C and 90°C. The tests were carried out with three replicates. Values followed by the same letter do not differ statistically according to the ANOVA and Tukey test at a 5% probability level.

The enzyme’s extremophile condition was not limited to an optimal strongly alkaline pH profile (pH 10.5) but also revealed itself at elevated temperatures. Lac_CB10 showed low relative activity (maximum 40%) with no significant increase between 37°C and 60°C; however, at 70°C, there was a significant increase in activity to 68%, followed by another significant increase at 80°C (93%) and 90°C, with the latter reaching its optimal temperature with a relative activity of 100%. (Fig. 5A).

Thermostability determination was carried out by incubating the enzyme for 5 h at 80°C and 90°C and measuring its activity on guaiacol substrate at its optimal pH. Lac_CB10 was thermostable at both temperatures, maintaining more than 50% of its activity for 5 h and reaching a relative maximum activity of 100% at 80°C after 270 min and at 90°C after 210 min. The prolonged activity of the enzyme at high temperatures is considered a vital parameter for meeting commercial requirements, as laccases with good thermotolerance properties are important for industrial processes.

Recently, our team conducted a study based on *in silico* data analysis of modeling and molecular docking for Lac_CB10 (31), and the main findings suggest that the enzyme belongs to the group of the family H—Bacterial CopA of laccases, which consists of a superfamily of enzymes that are potentially involved in greater copper resistance (1). These results, combined with our experimental data, suggest that these enzymes are important for the bioremediation of effluents and contaminated soils that commonly have extreme pH values and temperatures.

To evaluate the effect of inhibitors and ions on laccase activity, the optimal pH (CAPS pH 10.5) and ideal temperature (90°C) were used. Various inhibitors, including alcohols, ketones, and detergents such as SDS, Triton X-100, and Tween 80, were tested at concentrations of 0.5% and 1% (vol/vol); EDTA and DTT, at concentrations of 1 mM, 5 mM, and 10 mM; and methanol and acetone, at concentrations of 1%, 2%, and 5% (vol/vol). The results showed that all these inhibitors significantly reduced enzymatic activity, maintaining it below 30%, except for the detergent Triton X-100, which exhibited slightly greater activity (42%) than the other inhibitors, although it was still relatively low (Table 1).

**TABLE 1:** Influence of inhibitors on laccase enzymatic activity^*a*^

Inhibitor	Concentration	Relative activity (%)	
Methanol	1%	20.4 ± 0.1	c
Methanol	2%	25.5 ± 1.1	b
Methanol	5%	22.9 ± 1.8	c
Acetone	1%	15.6 ± 0.8	c
Acetone	2%	13.2 ± 0.8	c
Acetone	5%	15.9 ± 0.4	c
SDS	0.5%	14.5 ± 0.8	c
SDS	1%	16.2 ± 0.5	c
Triton X-100	0.5%	14.5 ± 0.17	c
Triton X-100	1%	42.0 ± 8.2	a
Tween 20	0.5%	15.1 ± 0.7	c
Tween 20	1%	18.2 ± 2.2	c
EDTA	1 mM	17.2 ± 1.6	c
EDTA	5 mM	19.2 ± 1.0	c
EDTA	10 mM	16.9 ± 0.7	c
DTT	1 mM	14.7 ± 0.4	c
DTT	5 mM	17.7 ± 1.8	c
DTT	10 mM	23.8 ± 22.7	c

^*a*^ Reactions were performed using SDS, Triton X-100, and Tween 80 at concentrations of 0.5% and 1% (vol/vol), EDTA and DTT at concentrations of 1 mM, 5 mM, and 10 mM, as well as methanol and acetone at 1%, 2%, and 5% (vol/vol). Lowercase letters indicate significant differences between each condition tested. Values followed by the same letter do not differ statistically, according to ANOVA and Tukey’s test at a 5% probability.

The evaluation of the CuSO_4_, MgSO_4_, NiCl_2_, CaCl_2_, NaCl, CoCl_2_, KCl, FeSO_4_, MnSO_4_, Al_2_(SO_4_)_3_, Li_2_SO_4_, AgNO_3_, BaCl_2_, ZnSO_4_, CdCl_2_, and KI ions indicated an increase in the relative activity of approximately 130% in the presence of Cu^2+^ and 122% in the presence of Cd^2+^. All the other ions tested did not significantly affect the activation of Lac_CB10. The Mn^2+^ ion had no significant impact on activity (70%), while the Ni^2+^ ion, on the other hand, exerted a strong inhibitory effect on enzymatic activity, reducing the relative activity to 13% (Table 2).

**TABLE 2:** Influence of ions on laccase enzymatic activity^*a*^

Ion	Relative activity (%)	
CuSO_4_	129.7 ± 20.8	a
MgSO_4_	43.2 ± 21.1	d
NiCl_2_	12.8 ± 2.1	h
CaCl_2_	44.3 ± 0.9	c
NaCl	16.8 ± 0.3	h
CoCl_2_	24.4 ± 2.8	e
KCl	20.9 ± 1.1	g
FeSO_4_	40.3 ± 3.0	e
MnSO_4_	69.6 ± 8.2	b
Al_2_(SO_4_)_3_	16.1 ± 0.8	h
Li_2_SO_4_	17.1 ± 0.2	h
AgNO_3_	21.7 ± 1.0	f
BaCl_2_	26.8 ± 0.5	e
ZnSO_4_	15.9 ± 0.3	h
CdCl_2_	121.9 ± 1.2	a
KI	21.0 ± 1.3	g

^*a*^ Reactions were carried out using CuSO_4_, MgSO_4_, NiCl_2_, CaCl_2_, NaCl_2_, CoCl_2_, KCl, FeSO_4_, MnSO_4_, Al_2_(SO_4_)_3_, Li_2_SO_4_, AgNO_3_, BaCl_2_, ZnSO_4_, CdCl_2_, and KI at a concentration of 2 mM. Lowercase letters indicate significant differences between each condition tested. Values followed by the same letter do not differ statistically, according to ANOVA and Tukey’s test at a 5% probability.

### Enzymatic degradation and scanning electron microscopy of Lignin

**Fig 6:**
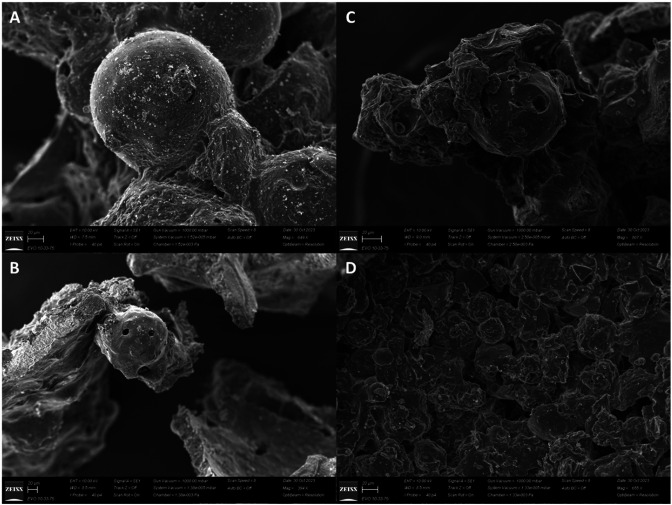
Scanning electron microscopy (SEM) images of alkalin lignin degradation. Treatment without inducer [(A) abiotic control and (B) treatment] and treatment with copper [(C) abiotic control and (D) treatment]. Experiments were conducted at 55°C for 32 h at 150 rpm.

The degradation of lignin by Lac_CB10 was analyzed after 32 h of treatment. The assay containing only the bacterial cell and lignin showed 16.33% degradation, while the assay containing copper as an inducer was able to degrade 52.27%. Scanning electron microscopy was used to verify the structural changes in lignin; abiotic treatments did not alter the structure of the lignin, while treatment without an inducer did not significantly change the structure (Fig. 6A and B). Treatment with copper caused the most structural changes, drastically altering the surface (Fig. 6C and D). These parameters are important since the use of inducers industrially increases the cost of the process; therefore, substituting expensive inducers like isopropyl-β-D-thiogalactopyranoside (IPTG) with copper, for more economical inducers, is advantageous, as was the case in this study. The fact that Lac_CB10 exhibits degradation capacity even in the absence of an inducer demonstrates that this enzyme indeed has the potential for lignin degradation.

## DISCUSSION

Aside from the “small laccases,” which have one fewer subunit, laccases generally possess four copper atoms per subunit, regardless of whether they are monomeric or composed of several chains (32). These copper atoms are divided into three cupric centers: Type 1 copper or blue copper (T1), which absorbs at 600 nm; Type 2 copper or normal copper (T2), which does not absorb in the visible spectrum; and Type 3 copper (T3) or binuclear coupled copper centers, which absorb light at approximately 330 nm (16).

Although it is a general characteristic of laccases and the MCO family to have these four copper atoms comprising the catalytic center, there are reports of characterized laccases in which copper atoms have been replaced by other molecules, such as the laccases from *P. ostreatus*, in which one copper atom was replaced by two zinc ions and one iron (33), and the laccases from *Trametes hirsuta*, in which one copper atom was replaced by manganese (34).

Based on these spectral characteristics, Lac_CB10 exhibited typical peaks of yellow laccases (8, 35) (Fig. 2B), such as the yellow laccase from *Bacillus licheniformis* described by Sharma et al. (21) (36), which displayed an absorption spectrum between 230 nm and 280 nm and lacked a peak at 600 nm; these characteristics are characteristic of the Type 1 copper center, which gives blue laccases their typical color. The absorption peak at approximately 300 nm corresponding to the spectral region of the Type 3 copper center is described in the literature as being responsible for making the enzyme solution colorless or yellow; hence, these laccases with unusual spectra are referred to as “white” or “yellow” laccases (5, 16).

However, despite exhibiting the spectral characteristics of yellow laccases, the enzymatic preparation of Lac_CB10 supplemented with copper had a blue coloration (Fig. 2C), even without showing an absorption peak in the UV‒Vis spectrum at 600 nm. A similar situation was reported for a bacterial laccase from *Stenotrophomonas maltophilia* AAP56 (SmLac), which also showed a blue color when copper was added but behaved like a yellow laccase in the UV‒Vis spectrum (37).

Similarly, Mot et al. (8) elucidated, through additional electronic paramagnetic resonance (EPR) analyses, the spectral characteristics of the fungal laccase from *S. sclerotiorum*, revealing that despite the characteristics of a yellow laccase in the UV‒vis spectrum, the EPR analysis also indicated peaks characteristic of a blue laccase.

It is known that laccases have different optimal pH values for different substrates due to the difference in the redox potential of Type 1 copper between laccase and the substrate, which increases with increasing pH, resulting in the transfer of electrons from the T1 center and increased activity at alkaline pH (38). For phenolic substrates such as guaiacol, the results are consistent with the literature, where laccases are reported to have higher activity at more basic pH values. The pH of the MSKLAC laccase from *Bacillus* sp. MSK-01 was tested on the substrates syringaldazine, ABTS, DMP, and guaiacol, which were 7.0, 4.5, 8.0, and 8.0, respectively, and was found to be highly stable between pH 5.0 and 10.0 for 24 h (16).

Intriguingly, regarding the activity of Lac_CB10 at high temperatures, as well as its thermostability, the enzyme was not expected to be secreted from thermophilic sources. However, Lac_CB10 is not the first thermophilic enzyme described for *Chitinophaga* sp. CB10, as our research group recently described a hyperthermophilic metallo-carboxypeptidase (39), highlighting its promise as a bacterial source for enzymes of industrial interest. Furthermore, the spectral data suggest that Lac_CB10 is likely a yellow laccase, which reinforces the high stability and functionality under extreme conditions reported in the literature for the still little-known “yellow laccases” (8).

Although not common, extremophilic conditions have also been reported for some bacterial blue laccases, such as those proposed from agro-industrial residues produced by *Bacillus aquimaris* AKRCo2, which exhibit thermal stability at 75°C with a half-life of 4 h when guaiacol is used as a substrate (40). Similarly, laccases from *Alcaligenes faecalis* have shown optimal activity at 80°C and thermal stability in the range of 70–90°C, where activity was measured by monitoring the oxidation of 2,6-dimethoxyphenol (2,6-DMP) (21). The stability of purified laccases at various temperatures demonstrates their potential for industrial application as alternatives to synthetic plastics, chemicals, and biofuels through processes that include biowaste (21).

Similarly, laccases obtained from the *Bacillus* sp. strain WT, *Bacillus* sp. PK4, *Thermobifida fusca*, *B. tequilensis* SN4, and *Geobacillus thermocatenulatus* (41–43) exhibited optimal activity in the range of 50–70°C but had a short half-life, rapidly losing activity at high temperatures. However, Lac_CB10 is capable of maintaining more than 50% of its activity for five hours at both tested temperatures (80°C and 90°C), which is highly important for industrial processes.

Treatment with copper as an inducer yielded unexpectedly satisfactory results. Moreover, the ability of Lac_CB10 to degrade lignin even in the absence of an inducer underscores the inherent potential of this enzyme for lignin degradation. These findings are significant because the use of inducers can increase the cost of industrial processes. Therefore, replacing these materials with more economical alternatives, such as copper in this study, is beneficial. These degradation results surpassed those reported in reference (30), where purified CotA-SL7 from *Bacillus altitudinis* achieved 31.2% degradation after 20 h at pH 5.0. This finding suggested that enzymatic activity could be further directed, especially when the enzyme is purified and used at its optimal pH. In contrast, Lac_CB10 was utilized within cells and at the cell growth pH rather than at the optimal pH for enzymatic activity, yet it still exhibited high degradation. This highlights the robustness of Lac_CB10 and its potential for efficient lignin degradation under less-than-ideal conditions.

### Conclusion

To the best of our knowledge, Lac_CB10 is the first yellow bacterial laccase described for the genus *Chitinophaga* that exhibits thermophilic and alkaline characteristics. These traits strongly suggest that this enzyme is highly promising for industrial processes, which are often conducted in high-temperature environments and extreme pH conditions. Lac_CB10 showed maximum activity at pH 10.5 with guaiacol as a substrate and remained stable, retaining more than 50% of its activity for 5 h at elevated temperatures (80°C and 90°C). This finding is consistent with the limited data available on yellow laccases and contributes to the establishment of this group as a promising target for biotechnological prospecting. The alkaline lignin assays indicated that Lac_CB10 is a highly promising enzyme for the degradation of this recalcitrant compound and could be effective as a biomass-degrading enzyme cocktail.

## Data Availability

Sequencing data are available on request.
